# Growth Rate-dependent Cell Death of Diatoms due to Viral Infection and Their Subsequent Coexistence in a Semi-continuous Culture System

**DOI:** 10.1264/jsme2.ME20116

**Published:** 2021-01-01

**Authors:** Yuji Tomaru, Haruo Yamaguchi, Takeshi Miki

**Affiliations:** 1 Hatsukaichi Field Station, Fisheries Technology Institute, Japan Fisheries Research and Education Agency, National Research and Development Agency, Maruishi, Hatsukaichi, Hiroshima 739–0452, Japan; 2 Faculty of Agriculture and Marine Science, Kochi University, Nankoku, Kochi 783–8502, Japan; 3 Ecology and Environmental Engineering, Faculty of Advanced Science and Technology, Ryukoku University, Yokotani, Seta Oe-cho, Otsu, Shiga, 520–2194, Japan

**Keywords:** diatom, population dynamics, viral infection

## Abstract

Viral infections are a major factor in diatom cell death. However, the effects of viruses on diatom dynamics remain unclear. Based on laboratory studies, it is hypothesized that virus-induced diatom mortality is dependent on the diatom growth rate. The present study aimed to elucidate the relationship between the diatom growth rate and virus-induced mortality using model systems of the marine planktonic diatom, *Chaetoceros tenuissimus* and its infectious viruses. We also examined the fate of diatom populations in a semi-continuous dilution culture system, in which host growth rates were controlled at 0.69, 2.08, and 3.47 day^–1^. Diatom populations gradually decreased following the viral inoculation of each culture system, and virus-induced mortality inversely correlated with the diatom growth rate. Furthermore, the viral burst size was slightly higher in lower growth rate cultures. These results suggested that the host physiological status related to the growth rate affected viral infection and proliferation. Diatom populations were not completely lysed or washed out in any of the dilution systems; they showed steady growth in the presence of infectious viruses. This may be partially explained by defective interference particles from viruses and cell debris. The present results indicate that diatoms in dilution environments maintain their populations, even under viral pressure. Moreover, diatom populations with a low growth rate may partially sustain higher growth populations through nutrient recycling following virus-induced cell death. The results of the present study provide insights into diatom dynamics in natural environments in the presence of infectious viruses.

Diatoms (Bacillariophyta) are unicellular, photosynthetic, eukaryotic algae found in aquatic environments worldwide. Their contribution to global biogeochemical cycling is significant (Tréguer *et al.*, 2018) because they account for a large part of marine primary production, comprising approximately 40% of ocean productivity (Field *et al.*, 1998). Recent studies on diatoms showed their metabolic and ecological significance in biogeochemical processes, including oxygen production, nutrient recycling, and carbon dioxide removal (Armbrust, 2009; Bowler *et al.*, 2010; Benoiston *et al.*, 2017). Therefore, diatom dynamics need to be elucidated in more detail to obtain a better understanding of the earth ecology.

Diatom population dynamics are principally affected by abiotic factors (Sarthou *et al.*, 2005); decreases are attributed to zooplankton grazing, sedimentation, and attacks by bacterial and fungal parasites (Amin *et al.*, 2012; Scholz *et al.*, 2016) and viruses (Tomaru *et al.*, 2015). Viral infections have been suggested as a major factor influencing the phytoplankton fate in nature (Mojica and Brussaard, 2014). Diatom viruses have a global distribution (Labonte and Suttle, 2013; Miranda *et al.*, 2016; Vlok *et al.*, 2019). Diatom virus abundance in natural waters specifically increases during diatom bloom periods and both populations may coexist for more than one month (Bettarel *et al.*, 2005; Tomaru *et al.*, 2011). However, the quantitative impact of viruses on natural diatom populations remains unclear, even in culture experiments. To obtain a clearer understanding of viral effects on diatom dynamics in nature, their relationship needs to be examined in culture experiments.

Laboratory experiments previously showed that in contrast to other algal host and virus systems, most diatom viruses cause host population crashes under stationary phases, whereas a few lead to rapid lysis during logarithmic growth phases (Tomaru *et al.*, 2015; Arsenieff *et al.*, 2019). Although the factors driving these phenomena are unknown, one hypothesis is that the virus-induced mortality of a diatom population depends on the diatom population growth rate. Therefore, the present study investigated the relationship between the growth rate of the marine planktonic diatom *Chaetoceros tenuissimus* Meunier and its virus-induced mortality using a semi-continuous culture method that enabled algal growth rate control. Viruses included the single-stranded DNA (ssDNA) virus *C. tenuissimus* DNA virus type-II (CtenDNAV-II) and the single-stranded RNA (ssRNA) virus *C. tenuissimus* RNA virus type-II (CtenRNAV-II) (Kimura and Tomaru, 2015). We also examined the fates of host–virus relationships in semi-continuous culture systems. The present results may provide a better understanding of diatom blooms in natural environments in the presence of infectious viruses.

## Materials and Methods

### Algal cultures and growth conditions

The axenic, clonal algal strain used in the present study, *C. tenuissimus* NIES-3715 (previous strain name: 2–10) (Shirai *et al.*, 2008), was isolated from Seto Inland Sea, Japan, on 10 August 2002. Strain NIES-3715 is sensitive to both* C. tenuissimus* DNA virus type-II (CtenDNAV-II) and *C. tenuissimus* RNA virus type-II (CtenRNAV-II) (Kimura and Tomaru, 2015), which were kept at the National Research Institute of Fisheries and Environment of Inland Sea, Japan. Algal cultures were maintained in SWM-3 medium (salinity 30) enriched with 2 nM Na_2_SeO_3_ (Imai *et al.*, 1996) under a 12:12 h light:dark cycle of approximately 800‍ ‍μmol of photons m^–2^ s^–1^ using white LED illumination at 25°C to gain a high growth rate of 3.47 day^–1^.

### Viral inocula

Exponentially growing cultures of *C. tenuissimus* NIES-3715 were inoculated with CtenDNAV-II and CtenRNAV-II (0.1% [v/v]), which were stored at 4°C in the dark, and incubated for 7‍ ‍d under the growth conditions described above. Lysates were passed through a 0.2-μm polycarbonate membrane filter (Whatman^®^ Nuclepore Track-Etched Membranes; Merck KGaA) to remove cellular debris and then stored at –80°C. Filtered lysates were used as experimental inocula. CtenDNAV-II and CtenRNAV-II titers after thawing were 3.47×10^9^±1.35×10^9^ infectious units mL^–1^ and 6.35×10^9^±3.32×10^9^ infectious units mL^–1^, respectively (average±standard deviation, *n*=4) (see section 2.5 Virus enumeration).

### Semi-continuous culture

A semi-continuous culture experiment was designed to estimate virus-induced mortality in the host populations growing at three different growth rates (1, 3, and 5 divisions day^–1^ corresponding to μ=0.69, 2.08, and 3.47 day^–1^, respectively). *C. tenuissimus* NIES-3715 was initially inoculated into a flask in a batch culture system and incubated under the conditions described above until cell concentrations increased to higher than 10^6^‍ ‍cells‍ ‍mL^–1^. These cultures were used in semi-continuous culture experiments. In these systems, 50.0% (low dilution, Ld), 87.5% (medium dilution, Md), and 96.9% (high dilution, Hd) of the cultures were replaced with fresh SWM-3 medium every 24 h. After 2‍ ‍d of the semi-continuous culture, viral suspensions were inoculated daily into the culture at the same time as medium replacement. In the first virus inoculation on day 2, the multiplicities of infection (MOI) in Ld, Md, and Hd cultures were 3.2–3.3, 1.9–1.9, and 4.1–4.2 for CtenDNAV-II, and 1.7–1.8, 1.7–1.7, and 3.4–3.7 for CtenRNAV-II, respectively. The inoculation volumes of the CtenDNAV-II and CtenRNAV-II suspensions were 0.2 and 0.1% v/v, respectively. A culture inoculated with fresh medium (no virus) served as a control. Each experiment was performed in duplicate. Residual cultures after medium replacement were used for cell counts and viral titrations. Cell counts were performed immediately with no fixation. The samples used for viral titer estimations (infectious units mL^–1^) were passed through 0.2-μm filters (DISMIC^®^-25CS020AS; Advantec) to remove cellular debris. Filtrates were stored at –80°C until analyzed. The titers of the viruses used in the present study may have been reduced because of the above-described preservation steps. However, the titers of diatom viruses are generally highly stable at low temperatures, even without flash freezing using liquid nitrogen (Shirai *et al.*, 2008; Tomaru and Nagasaki, 2011). Therefore, the results of the titers were not expected to affect the conclusions of the present study.

### Live cell counting

Live cell numbers were counted using an image-based cytometer (Tali^®^ Image Cytometer; Thermo Fisher Scientific) (Tomaru and Kimura, 2016). The SYTOX^®^ Green nucleic acid stain (excitation, 504 nm; emission, 523 nm; Thermo Fisher Scientific) was added to the cell suspension (1% [v/v]) at a final concentration of 0.5‍ ‍μM. A 25-μL aliquot of culture was placed on a disposable counting slide (Thermo Fisher Scientific). Cells were counted according to the manufacturer’s instructions after standing for 10‍ ‍min in the dark at room temperature to allow the cells to settle. The SYTOX^®^ Green stain only permeates cells with compromised plasma membranes, but does not cross the membranes of live cells. Cell counts were performed using the red (excitation filter, 543/22 nm; long-pass emission filter, 585 nm) and green (excitation filter, 466/40 nm; emission filter, 525/50 nm) channels of the image-based cytometer. The cell size range (in a bright field), red fluorescent threshold, green fluorescent threshold, circularity, and sensitivity were set at 3–20‍ ‍μm, 1,200, 1,200, 8, and 9, respectively. Cells with red fluorescence and no green fluorescence were counted as live cells. Cell counts were performed in duplicate for each sample.

### Virus enumeration

The number of infectious viral units was assessed using the extinction dilution method (Suttle, 1993). Briefly, stored filtrates were rapidly thawed at 25°C, and then diluted with SWM3 medium in a series of 10-fold dilution steps. Aliquots (100‍ ‍μL) of each dilution were added to 8 wells in cell-culture plates with 96 flat-bottomed wells and mixed with 150‍ ‍μL of an exponentially growing host algae culture. Cell culture plates were incubated at 20°C under a 12:12 h light:dark cycle of 130–150‍ ‍μmol photons m^–2^ s^–1^ (cool white fluorescent illumination) and cultures were monitored using an optical microscope (Nikon Ti) for up to 14‍ ‍d for culture lysis. The strong light intensity (section 2.1, 800‍ ‍μmol of photons m^–2^ s^–1^) adopted in growth experiments was not suitable to enumerate the virus titer. Under this condition, the growth of *C. tenuissimus* is too fast and results in rapid death even in a virus-free culture. Therefore, we used low light intensity to enumerate the virus titer in this culture-based method. Culture lysis due to viral infection was typically observed as the complete degradation of host cells in a well. Virus abundance was calculated from the number of wells in which algal lysis occurred using a BASIC programme as previously described (Nishihara *et al.*, 1986).

### Model formulation and parameter estimation

#### Exponential growth model for the semi-continuous culture system

A simple exponential growth model was developed to estimate host growth parameters and epidemic parameters. *N_k_* and *N_k_’* denoted host cell densities just before and after the exchange of growth media on day *k*, respectively. *N_k_’* was represented by the dilution series *j, d_j_*, as follows:

Nk'=(1-dj)Nk

 (1)

where (*d*_1_, *d*_2_, *d*_3_)=(0.500, 0.875, 0.969); each corresponded to 50, 87.5, and 96.9% dilution, respectively.

*T* denotes the time that passed after each daily dilution and *n_k_* (*T*) represents the population density at time *T* on day *k*. During the continuous culture phase of each day (0≤*T*<1) on day *k*, it was assumed that the host population density of scenario *j* followed a density-independent replication with a rate of *r_j_* (day^–1^) and density-independent, virus-induced mortality with a rate of *v_j_* (day^–1^). Under this assumption, population dynamics were given by:

$$\frac{dn_{k}(T)}{dT}=r_{j}n_{k}\left(T\right)-v_{j}n_{k}\left(T\right)=\left(r_{j}-v_{j}\right)n_{k}\left(T\right)\;0\leq T<1$$

 (2)

where *n_k_*(0)=*N_k_′* and $N_{k+1}=\;\lim_{T\to 1}\;n_{k}\left(T\right)$. Based on equations 1–2, the initial condition was calculated as:

$$n_{k}\left(0\right)=\left(1-d_{j}\right)\;\lim_{T\to 1}\;n_{k-1}\left(T\right)=\left(1-d_{j}\right)N_{k}$$

 (3)

#### Estimation of virus-induced mortality

The combination of equations 1–3 gave the following solution:

$$N_{k}={\left(1-d_{j}\right)}^{k}N_{0}e^{(r_{j}-v_{j})k}k=0,\;1,\;2,....$$

This was rearranged by logarithmic scaling to:

$$\ln N_{k}=\ln N_{0}+k\times \ln(1-d_{j})+(r_{j}-v_{j})k$$

$$=\ln N_{0}+\left[\ln(1-d_{j})+(r_{j}-v_{j})\right]k$$

 (4)

Here, the characteristics of semi-continuous culture systems, namely, the dilution rate and replication rate, need to be balanced when virus-induced mortality is not considered (*i.e.* culture without viral particles). This balance was represented by:

$$\ln(1-d_{j})+r_{j}=0$$

 (5)

Therefore, Eq. 4 was simplified as:

$$\ln N_{k}=\ln N_{0}-v_{j}k$$

 (6)

With Eq. 6 and the ln*N_k_* dataset, we estimated the virus-induced mortality *v_j_* (day^–1^) value using a simple linear regression. The data used to calculate virus-induced mortalities were those from 0–4 dpi for Ld and Md cultures for both viral treatments, and from 0–3 dpi and 0–5 dpi for Hd cultures inoculated with CtenDNAV-II and CtenRNAV-II, respectively.

#### Accumulated mortality of host cells

The method used to calculate the accumulated mortality of host cells each day (*i.e.* the total number of cells killed by viral lysis) was as follows: since the abundance of viruses, and, thus, virus-induced host mortality naturally changed during the semi-continuous culture, virus-induced mortality was estimated on each day, *k*, by

$$\ln(\frac{N_{k}}{N_{k-1}})=-v_{j,k}$$

 (7)

The initial condition on day *k* followed Eq. 3. During the incubation on day *k*, the population dynamics of host cells followed the following equation (c.f., Eq. 2):

$$\frac{dn_{k}(T)}{dT}=r_{j}n_{k}\left(T\right)-v_{j,k}n_{k}\left(T\right)$$

$$=\left(r_{j}-v_{j,k}\right)n_{k}\left(T\right)\;0\leq T<1$$

 (8)

The solution was given by:

$$n_{k}(T)=n_{k}(0)\exp\left[(r_{j}-v_{j,k})T\right]\;0\leq T<1$$

 (9)

The virus-induced mortality number during the differential time *dT* on day *k* was calculated as the following expression because the second term on the right-hand side of Eq. 8 represented virus-induced mortality:

$$v_{j,k}n_{k}(T)dT=v_{j,k}n_{k}(0)\exp\left[(r_{j}-v_{j,k})T\right]dT\;0\leq T<1$$

 (10)

This quantity (Eq. 10) was summed (integrated) from *T*=0 to *T*=1 so that the accumulated mortality of host cells DC_j,k_ during the day was derived as follows:

$$\text{D}{\text{C}}_{j,k}=\;\int_{T=0}^{T=1}v_{j,k}n_{k}\left(T\right)dT$$

$$=v_{j,k}n_{k}\left(0\right)\int_{T=0}^{T=1}\exp\left[\left(r_{j}-v_{j,k}\right)T\right]dT,0\leq T<1\;$$

 (11)

This was further calculated as:

$$\text{D}{\text{C}}_{j,k}=v_{j,k}n_{k}\left(0\right){\left[\frac{1}{r_{j}-v_{j,k}}e^{\left(r_{j}-v_{j,k}\right)T}\right]}_{0}^{1}$$

$$=\frac{v_{j,k}n_{k}\left(0\right)}{r_{j}-v_{j,k}}\left[e^{\left(r_{j}-v_{j,k}\right)}-1\right]$$

 (12)

The combination of Eq. 5 and Eq. 12 gave DC*_j,k_*, noting that the total mortality number (DC*_j,k_*) may be greater than the initial host number [*n_k_*(0)] because cells replicated at a rate of *r_j_*.

#### Burst size and transmission

The averaged burst size on day *k* *b_j,k_* was calculated using DC*_j,k_* and the number of newly-produced viral particles *V_j,k_* (calculated by the number of infectious viral units; the increased amount of infectious viral units during a 24-h incubation) as:

$$b_{j,k}=\frac{V_{j,k}}{\text{D}{\text{C}}_{j,k}}$$

 (13)

The transmission rate per viral particle (or viral-induced mortality per viral particle) was calculated as:

$$\beta_{j,k}=\frac{v_{j,k}}{V_{j,k}}$$

 (14)

## Results

### Host cell dynamics

**Fig. 1.**
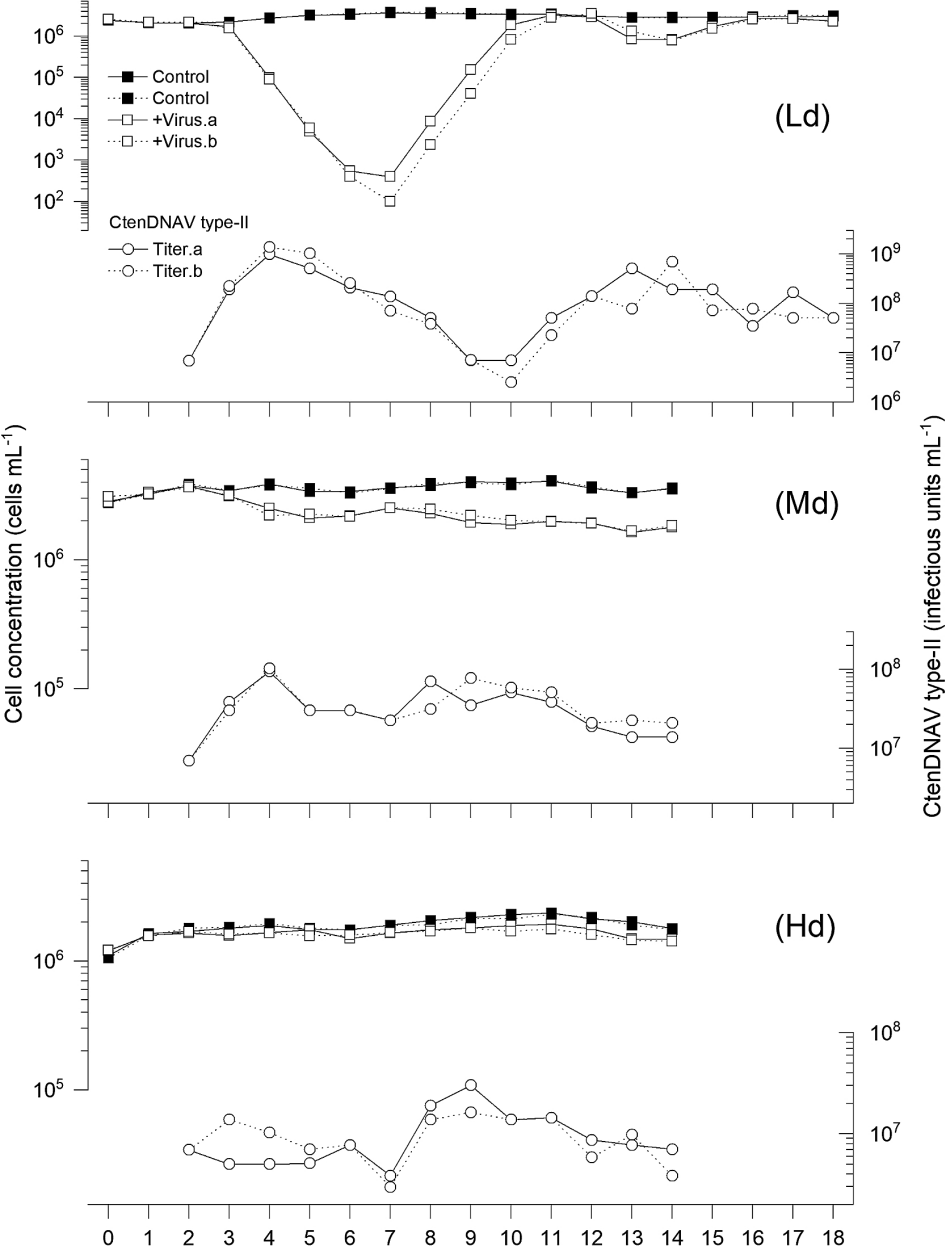
Changes in cell concentrations of CtenDNAV-II-infected or uninfected cultures of *Chaetoceros tenuissimus* strain NIES-3715 and viral titers under semi-continuous culture systems at low, medium, and high dilution rates: Ld) 0.500, Md) 0.875, and Hd) 0.969. Temporal changes in cell concentrations in the semi-continuous culture: uninfected culture (closed squares), CtenDNAV-II-inoculated culture (open squares), and CtenDNAV-II titers in the inoculated culture (open circles). Black and dotted lines are from duplicate semi-continuous culture experiments. Each of “a” and “b” following cell concentrations in the virus-inoculated culture and virus titer indicates the same data set from duplicate cultures. Virus titers on 0 dpi (day 2) indicate the final concentration after daily culture dilutions and viral inoculations. Thereafter (1 dpi, day 3), they indicate titers before culture dilutions, *i.e.* the results of a 24-h culture.

**Fig. 2.**
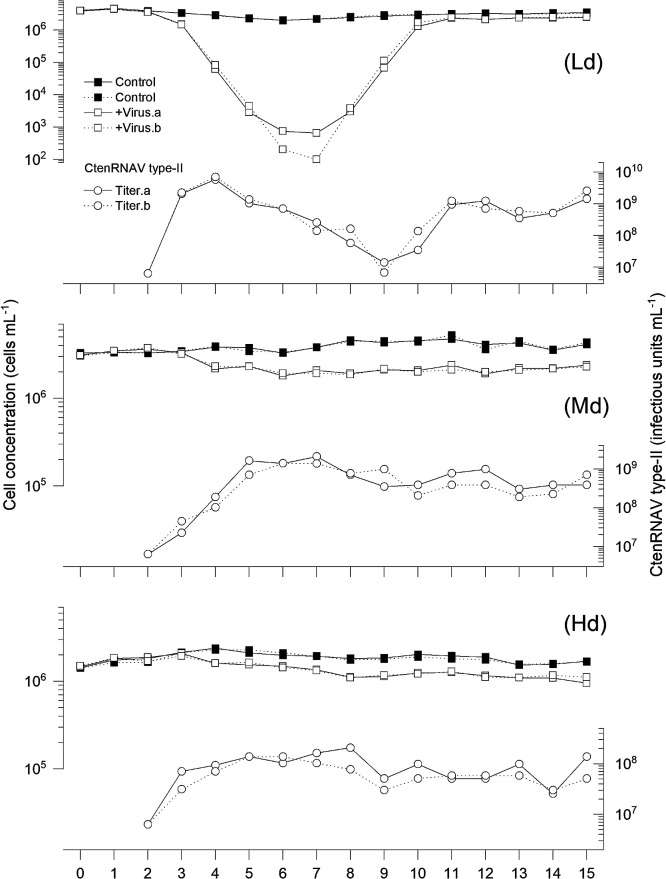
Changes in cell concentrations in CtenRNAV-II-infected or uninfected cultures of *Chaetoceros tenuissimus* NIES-3715 and viral titers under semi-continuous culture systems at different low, medium, and high dilution rates: Ld) 0.500, Md) 0.875, and Hd) 0.969. Temporal changes in cell concentrations in the semi-continuous culture: uninfected culture (closed squares), CtenRNAV-II-inoculated culture (open squares), and CtenRNAV-II titers in the inoculated culture (open circles). Black and dotted lines are from duplicate semi-continuous culture experiments. Each of “a” and “b” following cell concentrations in the virus-inoculated culture and virus titer indicates the same data set from the duplicate cultures. Virus titers on 0 dpi (day 2) indicate the final concentration after daily culture dilutions and viral inoculations. Thereafter (1 dpi, day 3), they indicate titers before culture dilutions, *i.e.* the results of a 24-h culture.

Cell concentrations in the semi-continuous culture systems for the Ld, Md, and Hd experiments without viral inoculation were stable during the experiments at ~10^6^‍ ‍cells‍ ‍mL^–1^ (Fig. 1 and 2).

### Ld culture (50% dilution)

**Fig. 3.**
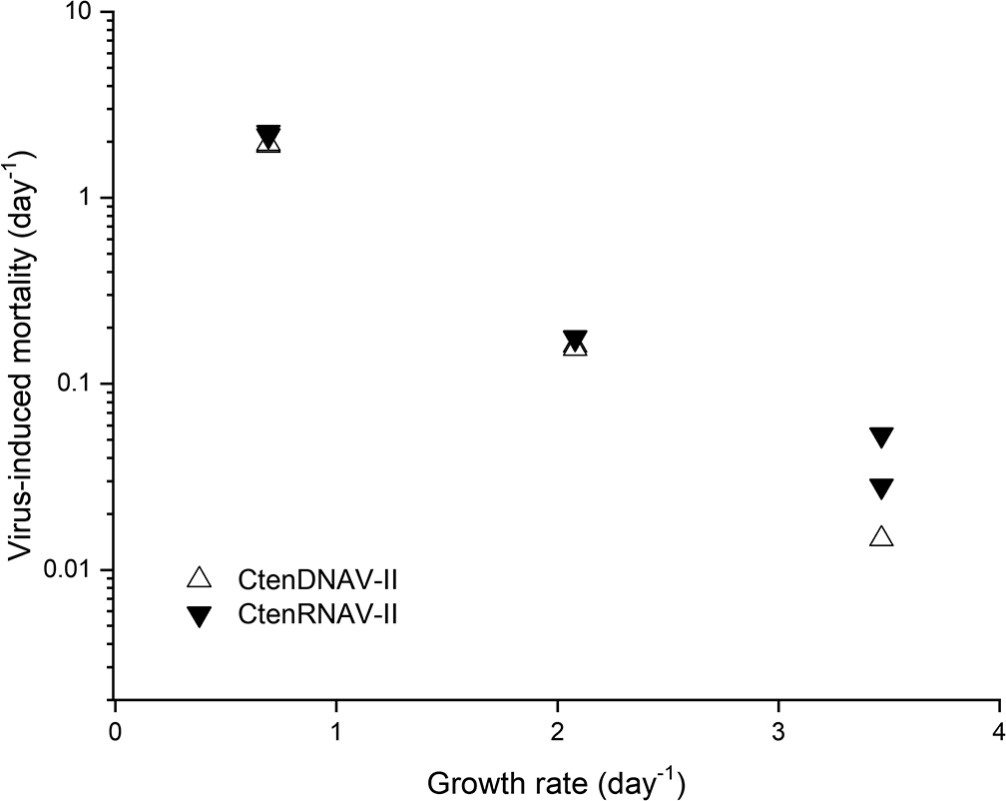
Correlation between growth rates of *Chaetoceros tenuissimus* NIES-3715 and virus-induced mortality due to the viral inoculation with CtenDNAV-II (open triangles) and CtenRNAV-II (closed triangles) in semi-continuous culture systems.

Cell concentrations in Ld cultures rapidly decreased after the viral inoculation with CtenDNAV-II and CtenRNAV-II (Fig. 1 and 2). Virus-induced mortalities until 4‍ ‍d post inoculation (dpi) in CtenDNAV-II- and CtenRNAV-II-inoculated cultures were 1.91–1.95 and 2.16–2.25 day^–1^, respectively (Fig. 3). Host cell concentrations in both the CtenDNAV-II- and CtenRNAV-II-inoculated cultures decreased to <10^3^‍ ‍cells‍ ‍mL^–1^ by day 7, and then rapidly increased again for 3 days and reached >10^6^‍ ‍cells‍ ‍mL^–1^ even in the presence of at least >10^7^ infectious units mL^–1^ (Fig. 1). During regrowth periods, the diatom growth rates in DNA and RNA virus-inoculated cultures were 3.19–3.85 and 2.22–4.15 day^–1^, respectively (Fig. 1 and 2). After rapid regrowth, cell concentrations in CtenDNAV-II-inoculated cultures showed a slight oscillation of 0.81×10^6^–3.21×10^6^‍ ‍cells‍ ‍mL^–1^ (Fig. 1A) and those in CtenRNAV-II-inoculated cultures reached >10^6^‍ ‍cells‍ ‍mL^–1^ (Fig. 2A). These concentrations were lower than those in the controls in most cases, at 30–>100% and 65–77% of the controls, respectively.

CtenDNAV titers in Ld cultures rapidly increased until 2‍ ‍dpi, reached 10^9^ infectious units mL^–1^, and then gradually decreased to <10^7^ infectious units mL^–1^, at which host cultures showed regrowth after viral inoculation (Fig. 1A). CtenDNAV-II titers increased again and oscillated when host cell cultures showed stable growth after day 10 (Fig. 1A). The dynamic pattern of CtenRNAV-II was similar to that of CtenDNAV-II.

### Md culture (87.5% dilution)

The cell concentration decrease estimated in CtenDNAV-II- and CtenRNAV-II-inoculated cultures was more moderate in Md cultures than in Ld cultures (Fig. 1 and 2). Virus-induced mortalities until 4 dpi were 0.15–0.16 day^–1^ in CtenDNAV-II-inoculated cultures and 0.18 day^–1^ in CtenRNAV-II-inoculated cultures (Fig. 1 and 2). Cell concentrations in virus-inoculated cultures stabilized after a slight decrease. Cell concentrations in CtenDNAV-II- and CtenRNAV-II-inoculated cultures ranged between 1.65×10^6^–2.53×10^6^‍ ‍cells‍ ‍mL^–1^ and 1.90×10^6^–2.33×10^6^‍ ‍cells‍ ‍mL^–1^, respectively. These abundances were roughly 42–70% lower than those of the controls.

The abundances of both CtenDNAV-II and CtenRNAV-II increased until 2 or 3 dpi, and then fluctuated at 10^7^ infectious units mL^–1^ and 10^8^ infectious units mL^–1^, respectively (Fig. 1 and 2).

### Hd culture (96.9% dilution)

The dynamic patterns of cell concentrations in Hd cultures were similar to those in Md cultures; however, the decrease in cell concentrations was more moderate (Fig. 1 and 2). In one of the two CtenDNAV-II-inoculated cultures, a cell concentration decrease was not observed until 3 dpi; therefore, mortality was not calculated. Virus-induced mortality was 0.01 day^–1^in CtenDNAV-II-inoculated cultures until 3 dpi and 0.03–0.05 day^–1^ in CtenRNAV-II-inoculated cultures until 5 dpi (Fig. 1B and 2B). Cell concentrations in CtenDNAV-II- and CtenRNAV-II-inoculated cultures were 1.45×10^6^–1.84×10^6^‍ ‍cells‍ ‍mL^–1^ and 1.90×10^6^–2.33×10^6^‍ ‍cells‍ ‍mL^–1^, respectively. Concentrations were mostly stable and constantly lower than those of control cultures (82 and 65% of the respective controls; Fig. 1).

CtenDNAV-II titers gradually decreased until 5 dpi. Higher CtenDNAV-II titers over the daily inoculated viral abundance (final concentration of 6.94×10^6^ infectious units mL^–1^) were only observed from days 8 to 11 (1.38×10^7^ infectious units mL^–1^ to 2.3×10^7^ infectious units mL^–1^, respectively). In CtenRNAV-II-inoculated cultures, virus titers gradually increased after the start of the inoculation. They remained at 10^8^ infectious units mL^–1^ from 3 to 6 dpi, then decreased and fluctuated in the order of 10^7^ infectious units mL^–1^ (Fig. 1).

### Virus-induced mortality

A regression analysis showed that virus-induced mortality in CtenDNAV-II- and CtenRNAV-II-inoculated cultures inversely correlated with the growth rate (r=0.999 [*n*=5] and 0.983 [*n*=6], respectively) (Fig. 3 and S1). Virus-induced mortality due to the viral inoculation was lower in higher dilution cultures.

### Burst size

Burst sizes were calculated based on Eq. 13. Until 2 dpi, CtenDNAV-II burst sizes in Ld cultures were 5.42×10^2^–1.39×10^3^ infectious units cell^–1^, which were higher than those in Md (1.19×10^2^–3.34×10^2^ infectious units cell^–1^) and Hd cultures (3.34×10^2^ infectious units cell^–1^; Table 1). Similarly, CtenRNAV-II burst sizes in Ld cultures (1.34×10^3^–6.87×10^3^ infectious units cell^–1^) were higher than those in Md (1.23×10^2^–4.04×10^2^ infectious units cell^–1^) and Hd cultures (6.99×10^2^–7.30×10^2^ infectious units cell^–1^; Table 1).

### Transmission rate

Transmission rates were calculated based on Eq. 14. The transmission rates of CtenDNAV-II in Ld and Md cultures did not significantly differ at >2×10^–8^ mL day^–1^ infectious unit^–1^, while those of Hd cultures were lower (<1×10^–8^ mL day^–1^ infectious unit^–1^; Table 2). In Ld cultures, CtenRNAV-II transmission rates at 0–1 dpi (13.8×10^–8^–14.5×10^–8^ mL day^–1^ infectious unit^–1^) were one order of magnitude higher than those of Md and Hd cultures, but not at 1–2 dpi (Table 2). The transmission rates of both viruses in Ld cultures were slightly higher at 0–1 dpi than at 1–2 dpi (Table 2).

## Discussion

The results of diatom population dynamics in low dilution cultures after inoculation with either virus appeared to consist of three different phases: (1) a period of decreasing cell concentrations after the viral inoculation, (2) the regrowth of diatom populations with the virus present, and (3) the stable coexistence of diatoms and viruses in semi-continuous culture systems. These three phases are discussed in the following sections.

### Virus-induced mortality and host growth rate

A negative relationship has been reported between diatom growth and viral infection. Diatom populations are nearly unaffected by viral inoculations in logarithmic growing phases, but quickly decrease during stationary phases (Tomaru *et al.*, 2014; Arsenieff *et al.*, 2019). The negative relationship between the cell growth rate and virus-induced mortality observed in the present study (Fig. 3) strongly supports the conclusions from previous studies. Moreover, burst sizes in the present study were slightly higher in lower dilution cultures, particularly for the RNA virus (Table 1); the physiological state of lower growth diatom cells may be preferable for viral replication.

In many marine microbial host–virus systems, positive interactions have been reported between host growth rates and virus-induced cell death with higher burst sizes (Middelboe, 2000; Mojica and Brussaard, 2014). In chemostat cultures, *Pseudoalteromonas* sp. mortality due to viral infections increases with higher dilution rates (Middelboe, 2000). The replication of *Chlorella* virus PBCV-1 was enhanced in actively growing host cells (Van Etten *et al.*, 1983). The bloom-forming raphidophyte *Heterosigma akashiwo* is sensitive to infection by its infectious virus (HaV01) during the host logarithmic growing phase, but becomes resistant in the stationary phase (Nagasaki and Yamaguchi, 1998). The present results do not provide a reason for the different diatom–virus relationships from those in other microalga-virus systems. We speculate that the physiological state related to the diatom growth rate may be an important factor influencing virus infection process efficiency.

The negative relationship between the diatom growth rate and virus-induced cell death may be important from the viewpoint of diatom survival in nature. Lower growth rate diatoms may be rapidly lysed due to viral infections, which enhances nutrient recycling through microbial degradation. We speculate that the nutrients released through this recycling process may sustain the survival of the higher growth rate population.

### Diatom population regrowth with a viral presence

After cell concentrations in Ld cultures reached a minimum, they rapidly increased again, even with a viral presence of at least >10^7^ infectious units mL^–1^ (Fig. 1 and 2). Diatom cells were not completely lysed and did not disappear in Ld cultures. To explain the regrowth of the *C. tenuissimus* populations under these high viral concentration environments, several hypotheses have been proposed.

The specific growth of mutant cells in the host population after the viral inoculation was previously observed in prokaryotes and eukaryotes. Resistant clones occurring in chemostats during the growth phase of a virus-sensitive *Pseudoalteromonas* sp. clone replaced sensitive host cells after lysis following the viral inoculation in a continuous culture system (Middelboe, 2000). Similarly, virus-resistant mutant strains of *Micromonas pusilla* (Mamiellaceae) and *Ostreococcus tauri* (Bathycoccaceae) have often been observed in their host–virus systems (Waters and Chan, 1982; Zingone *et al.*, 2006, Thomas *et al.*, 2011). *Heterocapsa circularisquama* (Dinophyceae) resistance to its RNA virus (HcRNAV) is attributed to its phenotypic plasticity. The system is not driven by a mutation, but by the intracellular suppression of viral genome replication that is only maintained in the presence of the virus (Tomaru *et al.*, 2009). After the dilution experiments in the present study ended (*i.e.* batch culture conditions), diatom populations that survived the viral inoculations showed rapid decreases and complete culture crashes within one week (data not shown), presumably because of viral infections. A similar dynamic pattern was previously reported (Tomaru *et al.*, 2014). Therefore, surviving *C. tenuissimus* cells in the present semi-continuous culture systems may not be mutant or *H. circularisquama*-like resistant cells.

Defective interfering particles (DIPs) are mutant viruses with partially deleted genomes that spontaneously arise during viral proliferation (Roux *et al.*, 1991; Thompson *et al.*, 2009). DIPs require coinfection with a wild-type virus to replicate within host cells. They inhibit the production of normal wild-type viruses and lytic infections (Thyrhaug *et al.*, 2003; Brown and Bidle, 2014). CtenRNAV-II abundances in titer only explain 8–42% of qPCR copy numbers (Tomaru and Kimura, 2016). In the present study, RNA virus copy numbers assessed with the qPCR assay were 2–175-fold higher than the number of infectious viruses estimated using the extinction dilution method (Fig. S2), which may partially support the existence of diatom virus DIPs. The underestimation or lower virus titers may be partially explained by host cell-derived molecules. Cells lysed by viral infections may provide cell debris other than progeny viruses and DIPs. These cell molecules may be specifically or non-specifically attached to infectious viruses and decrease viral abundance (Thyrhaug *et al.*, 2003). Furthermore, in bacteria and virus relationships, membrane vesicles produced by the host cells act as a decoy to trap viruses (Biller *et al.*, 2014). Therefore, a higher abundance of defective viruses and cell debris may be included in Ld cultures, inhibiting successful viral infection and enhancing the subsequent survival and growth of *C. tenuissimus* cells.

During Ld culture regrowth, the growth rate ranged from 2.22–4.15 day^–1^, which corresponded to approximately 3–6 divisions day^–1^. Therefore, most cells in the re-growing populations of Ld cultures may not be susceptible to viral infection, as discussed above. During regrowth, fast cell growth, DIPs, and cell debris may protect cells from viral infection.

### Virus-diatom coexistence

After Ld culture regrowth and the gradual decrease in host cells in higher dilution cultures, the diatom and virus populations appeared to stably fluctuate in the semi-continuous culture systems (Fig. 1 and 2). This result indicated that most cells in host populations were not infected and grew in culture, whereas a percentage was infected and contributed to viral proliferation. As discussed above, a faster growth rate, DIPs, and cell debris may positively affect host population survival and growth. Host cell concentrations in virus-inoculated cultures were lower than those in control cultures; this may be the result of balancing host growth, cell death, and infectious virus proliferation with the simultaneous increase in DIPs and debris that arose in each culture system. Virus-induced cell death may act as a self-control factor maintaining host–virus coexistence in each dilution environment.

Middelboe (2000) demonstrated the coexistence of bacteria and its virus in a continuous culture system. The cell concentrations of host bacteria coexisting with the virus in culture were lower than those in the virus-free control culture, similar to the results of the present study. The cultures contained two bacterial phenotypes, a higher growth rate virus-susceptible phenotype and a lower growth resistant phenotype; differences in cell yields were explained by the dominance of the low growth rate virus-resistant phenotype. As discussed above, virus-resistant mutant diatom cells may not have arisen during the study period. However, we cannot completely deny the possibility that some diatom cells were tentatively virus-resistant or in an unsusceptible condition during the semi-continuous culture. For example, the RNA virus resistance of *H. circularisquama* is derived from a block of virus genome replications in the cell and occurs via phenotypic plasticity (Tomaru *et al.*, 2009).

Thomas *et al.* (2011) demonstrated chronic infection-like eucaryotic algal host and virus coexistence. In that system, *O. tauri*-resistant clones produced infectious double-stranded DNA virus OtV particles with their growth. Virus coexisting diatom populations in batch systems after dilution experiments showed complete crashes within one week (data not shown). Although we cannot exclude this possibility, this phenomenon may not support the chronic infection-like relationship in the diatom host and virus systems.

The existence of spatial or physiological refuges from viral attacks for sensitive host cells may contribute to the stabilization of host–virus dynamics (Lenski, 1988). Thyrhaug *et al.* (2003) suggested that cell cycle-dependent virus production in *Pyramimonas orientalis* cultures infected with its infectious dsDNA virus (PoV) allowed host–virus system coexistence. However, we cannot fully explain what the refuge was in this system. In the present study, faster growth, DIPs, and debris produced from lysed cells may be possible refuges. Although the underlying coexistence mechanisms have not yet been revealed, the physiological status of the host cell is an important factor affecting diatom–virus interactions.

### Conclusion and implications

In the present study, the host growth rate inversely correlated with virus-induced mortality. The host physiological state related to the growth rate may have affected viral proliferation, including burst size. The present results suggest important ecological phenomena; however, the data available in this study are limited. Similar repetitive experiments may be needed to validate our conclusion in future studies. In addition, we were unable to elucidate the mechanisms governing the relationship between host population growth rates and viral infections. Further physiological studies at the single-cell level are needed to better understand the diatom–virus system in nature.

*C. tenuissimus* was able to maintain growth, even in the presence of viruses. Of note, this only happened when appropriate dilutions were performed (≥87.5% in the present study) and the necessary nutrient supplies were provided to support population growth. Low growth rate populations in natural environments may partially contribute to nutrient recycling through virus-induced cell lysis followed by microbial degradation, which, in turn, benefits the survival of higher growth rate cells. *C. tenuissimus* often blooms in coastal areas for more than one month in the presence of viruses (Tomaru *et al.*, 2011; 2018). The present results support the long-term coexistence observed in natural environments and may contribute to our understanding of the mechanisms underlying global primary production sustained by diatoms. Further studies on diatom–virus relationships that focus on the mechanisms of viral infection and stable diatom–virus coexistence at the molecular level will improve our understanding of *C. tenuissimus* dynamics in its natural environment.

## Citation

Tomaru, Y., Yamaguchi, H., and Miki, T. (2021) Growth Rate-dependent Cell Death of Diatoms due to Viral Infection and Their Subsequent Coexistence in a Semi-continuous Culture System. *Microbes Environ ***36**: ME20116.

https://doi.org/10.1264/jsme2.ME20116

## Supplementary Material

Supplementary Material

## Figures and Tables

**Table 1.** Burst sizes (infectious units cell^–1^) of CtenDNAV-II and CtenRNAV-II in different dilution cultures.

Virus	dpi	Semi-continuous culture, dilution rate^a^					
		Ld, 0.500		Md, 0.875		Hd, 0.969	
CtenDNAV-II	0–1	7.13×10^2^	5.42×10^2^	1.26×10^2^	1.19×10^2^	nd	3.34×10^2^
	1–2	8.81×10^2^	1.39×10^3^	3.34×10^2^	2.44×10^2^	nd	nd
CtenRNAV-II	0–1	1.34×10^3^	1.58×10^3^	1.23×10^2^	1.66×10^2^	nd	nd
	1–2	5.47×10^3^	6.87×10^3^	4.04×10^2^	2.62×10^2^	6.99×10^2^	7.30×10^2^

^a^ The two values in each column are from duplicate semi-continuous culture experiments. dpi, days post infection; Ld, low dilution; Md, medium dilution; Hd, high dilution; CtenDNAV-II, *Chaetoceros tenuissimus* DNA virus type-II; CtenRNAV-II, *C. tenuissimus* RNA virus type-II; nd, not detected

**Table 2.** Transmission rates (×10^–8^ mL day^–1^ infectious unit^–1^) of CtenDNAV-II and CtenRNAV-II in different dilution cultures.

Virus	Dpi	Semi-continuous culture, dilution rate^a^					
		Ld, 0.500		Md, 0.875		Hd, 0.969	
CtenDNAV-II	0–1	2.76	4.47	2.59	2.02	0.63	0.66
	1–2	2.80	2.39	1.85	3.38	nd	nd
CtenRNAV-II	0–1	14.5	13.8	1.45	2.55	nd	nd
	1–2	0.30	0.25	4.58	2.67	3.01	2.52

^a^ The two values in each column are from duplicate semi-continuous culture experiments. dpi, days post infection; Ld, low dilution; Md, medium dilution; Hd, high dilution; CtenDNAV-II, *Chaetoceros tenuissimus* DNA virus type-II; CtenRNAV-II, *C. tenuissimus* RNA virus type-II; nd, not detected
