# Supplementary material for: Growth Rate-dependent Cell Death of Diatoms due to Viral Infection and Their Subsequent Coexistence in a Semi-continuous Culture System
Source: Microbes Environ. 2021 Jan 1;36(1):ME20116. doi: 10.1264/jsme2.ME20116 (PMC7966941; doi:10.1264/jsme2.ME20116)
Supplement: Supplementary file 1 — Supplementary Material [file 36_20116_s1.pdf]

Supplemental information

	mortality	
Growth	DNA virus	RNA virus
0.70	1.91	2.16
0.70	1.95	2.25
2.08	0.16	0.18
2.08	0.15	0.18
3.47	Nd.	0.05
3.47	0.01	0.03

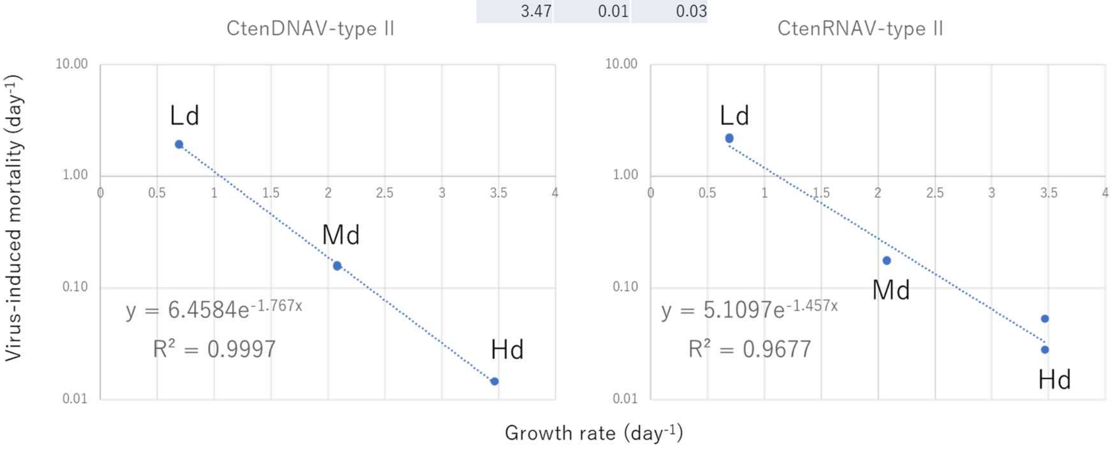

Fig. S1. Regression analysis for the relationships between diatom growth rates and virus-induced mortalities due to the viral inoculations shown in Fig. 3.

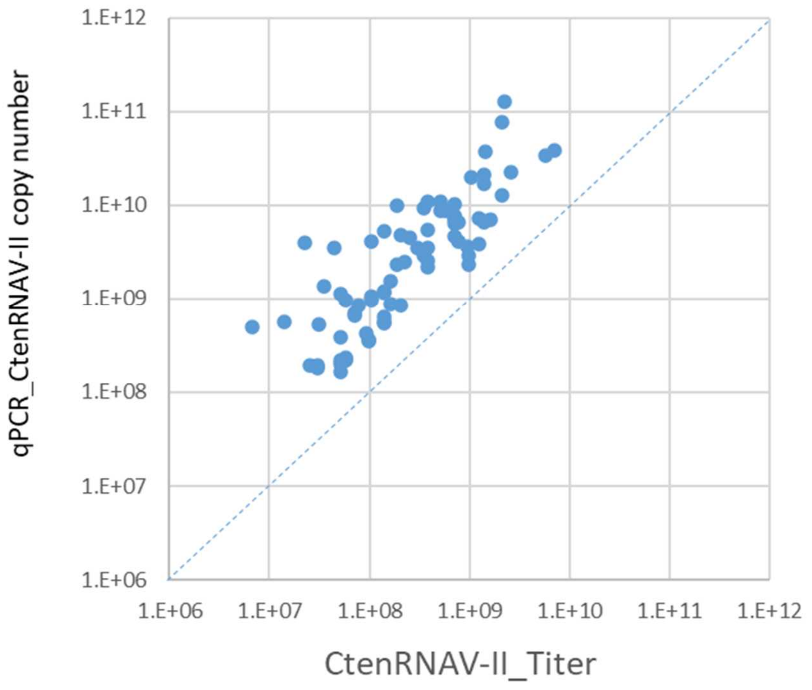

Fig. S2. Comparisons of virus numbers between the extinction dilution method (viral titer, see 'Materials and methods') and qPCR (viral copy number, Tomaru and Kimura 2016) in the CtenRNAV type-II inoculated experiments. The dotted line indicates where the titer: qPCR ratio is 1:1.

Literature Cited

Tomaru Y, Kimura K (2016) Rapid quantification of viable cells of the planktonic diatom *Chaetoceros tenuissimus* and associated RNA viruses in culture. Plankton Benthos Res 11:9-16
